# Attenuation of NLRP3 Inflammasome by Cigarette Smoke is Correlated with Decreased Defense Response of Oral Epithelial Cells to *Candida albicans*

**DOI:** 10.2174/1566524023666230612143038

**Published:** 2023-07-17

**Authors:** Fan Huang, Ruiqi Xie, Ruowei Li, Liu Liu, Maomao Zhao, Qiong Wang, Weida Liu, Pei Ye, Wenmei Wang, Xiang Wang

**Affiliations:** 1 Department of Oral Medicine, Nanjing Stomatological Hospital, Affiliated Hospital of Medical School, Nanjing University, Nanjing, China;; 2 Jiangsu Key Laboratory of Molecular Biology for Skin Disease and STIs, Department of Mycology, Institute of Dermatology, Chinese Academy of Medical Sciences (CAMS) & Peking Union Medical College (PUMC), Nanjing, China

**Keywords:** NLRP3 inflammasome, cigarette smoke, *Candida albicans*, oral epithelial cells, signal pathway, host defense

## Abstract

**Background:**

It is well recognized that both smoke and *Candida* infection are crucial risk factors for oral mucosal diseases. The nucleotide-binding domain-like receptor family pyrin domain containing 3 (NLRP3) inflammasome and its downstream effectors, interleukin (IL)-1β and IL-18, are pivotal to the host defense against *Candida* and other pathogens.

**Methods:**

The present study was designed to explore the effects of cigarette smoke and *C. albicans* on the NLRP3 inflammasome and its downstream signal pathway *via in vitro* cell model. Oral epithelial cells (Leuk-1 cells) were exposed to cigarette smoke extract (CSE) for 3 days and/or challenged with *C. albicans*.

**Results:**

Microscopically, Leuk-1 cells exerted a defense response to *C. albicans* by markedly limiting the formation of germ tubes and microcolonies. CSE clearly eliminated the defense response of Leuk-1 cells. Functionally, CSE repressed NLRP3 inflammasome, and IL-1β and IL-18 activation induced by *C. albicans* in Leuk-1 cells.

**Conclusion:**

Our results suggested that in oral epithelial cells, the NLRP3 inflam-masome might be one of the target pathways by which CSE attenuates innate immunity and leads to oral disorders.

## 1 INTRODUCTION

It is well known that cigarette smoke exposure is one of the major risk factors for systemic diseases [1]. In addition, it is also an increasingly well-recognized risk for oral diseases, such as oral leukoplakia, oral cancer, oral candidiasis, and periodontitis [1, 2]. Cigarette smoking has been known to increase susceptibility to infection, likely *via* dysregulation of immune function [3]. The oral cavity is an entrance into the human body, and it is usually the first region exposed to tobacco smoke [4, 5]. The oral mucosal epithelium acts as a crucial defense shield in the host's innate immune system, interacting with harmful stimulatory factors, such as smoke, and producing immune-related proteins, cytokines, and other defense molecules [6-8].

**Fig. (1):**
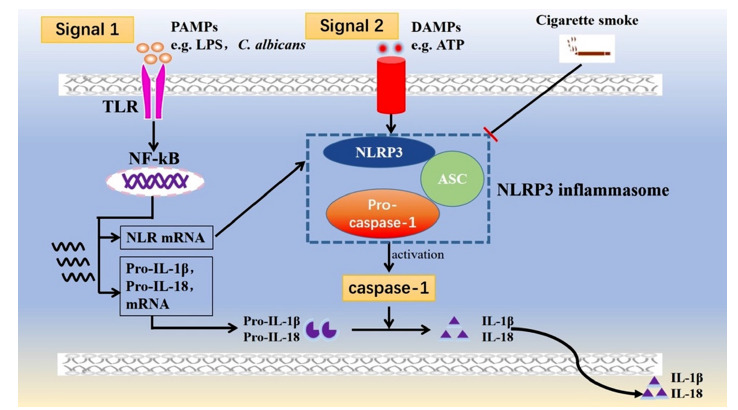
Schematic of NLRP3 inflammasome activation and the suppressive effect of cigarette smoke. Both signal 1 and signal 2 are required for NLRP3 inflammasome activation. Signal 1 is also known as the priming signal; various PAMPs, such as LPS and pathogens, “prime” the inflammasome by activating a TLR, thus inducing NF-kB activation leading to increased mRNA levels of pro-IL-1β, pro-IL-18, and NLRP3. Signal 2, also known as the activating signal, is mediated by stimulation with numerous DAMPs, such as ATP, and promotes the assembly of NLRP3, ASC, and pro-caspase-1, leading to the activation of the NLRP3 inflammasome complex. Activation of the NLRP3 inflammasome causes the activation of caspase-1, which cleaves the precursor forms of IL-1β and IL-18 into the corresponding mature forms, which are then secreted from the cell. LPS+ATP is the classical agonist of the NLRP3 inflammasome. However, according to the results of previous studies and our experiment, the addition of cigarette smoke can inhibit the expression of NLRP3, caspase-1, IL-1β, and IL-18. **Abbreviations:** PAMPs, pathogen-associated molecular patterns; DAMPs, danger-associated molecular patterns; LPS, lipopolysaccharides; ATP, adenosine triphosphate; TLRs, toll-like receptors; NF-kB, nuclear factor kappa-light-chain-enhancer of activated B cells; NLRP3, nucleotide-binding domain (NOD)-like receptor family pyrin domain containing 3; ASC, apoptosis-associated speck-like protein containing a C-terminal caspase recruitment domain.

Cytoplasmic pattern recognition receptors (PRRs) are a part of the innate immune system in the oral mucosal epithelium. These PRRs can recognize various pathogen-associated molecular patterns (PAMPs) or danger-associated molecular patterns (DAMPs) and activate host responses against pathogens or harmful substances [9]. Among the various PRRs, the NLRP3 inflammasome, the most fully characterized inflammasome to date, is composed of the sensor protein nucleotide-binding domain-like receptor family pyrin domain containing 3 (NLRP3), the adaptor protein apoptosis-associated speck-like protein (ASC), and the pro-inflammatory protein (caspase-1) [10]. NLRP3 inflammasome is involved in a lot of systemic diseases, for example, diabetes and cardiovascular diseases [11]. Several studies have also indicated that the NLRP3 inflammasome plays a key role in the pathogenesis of several common oral diseases, including periodontitis, dental pulp disease, oral lichen planus, and oral cancer, by mediating inflammatory and adaptive immune responses [12, 13]. To date, it has been commonly recognized that activation of the NLRP3 inflammasome depends on two signals: a priming signal, which is required for the upregulation of NLRP3, pro-IL-1β and pro-IL-18, and a second signal that triggers the assembly of the NLRP3 inflammasome complex. Upon NLRP3 inflammasome activation, active caspase-1 is cleaved, and IL-1β and IL-18 are subsequently released, which play an important role in the host defense against infection [14, 15, 16] (Fig. **1**).

As one of the most common commensal microbes in the human oral cavity, *Candida albicans* (*C. albicans*) can become an opportunistic organism if the flora is disturbed [17, 18]. *C. albicans* is highly polymorphic. Under certain conditions, original yeast cells can switch to the germ tubes and hyphae then in a few hours. In about 24 hours, dispersed micro-colonies of different sizes could be seen under the microscope or on a solid medium. Clinically, *C. albicans* has been considered a potential aetiological agent for potentially malignant oral disorders, such as oral leukoplakia and oral lichen planus, for example [18].

According to various studies, smoking impairs the innate immune defense of local mucosa and promotes the growth of *C. albicans*. A previous study reported the infection rate of *C. albicans* in smokers to be as high as 70%, while that in non-smokers to be only 30% [19]. Another study demonstrated that cigarette smoke promotes *C. albicans* adhesion, growth, and biofilm formation [20]. Our previous study indicated long-term cigarette smoking to suppress NLRP3 inflammasome activation and attenuate host defense in a rat model [21]. However, the impacts of cigarette smoke exposure and *C. albicans* infection on inflammasomes and defense effectors in oral epithelial cells have not been investigated to date. In particular, the specific mechanism by which cigarette smoke decreases inflammasome expression and the defense response of oral epithelial cells to *C. albicans* is still unclear. Thus, the present study aimed to characterize the combined effects of cigarette smoke exposure and *C. albicans* infection on the expression of the NLRP3 inflam-masome and its downstream effectors in oral epithelial cells, and to explore the potential mechanisms.

## 2 MATERIALS AND METHODS

### 2.1 Reagents

The goat anti-NLRP3 antibody (ab4207), the mouse anti-IL-1β antibody (ab8320), the rabbit anti-*C. albicans* antibody (ab53891), and Cy3-conjugated goat anti-mouse secondary antibody were purchased from Abcam (Cambridge, UK); the mouse anti-caspase-1 antibody (14F468) was purchased from Santa Cruz (California, USA); the mouse anti-IL-18 antibody (60070-1-Ig) and FITC-conjugated goat anti-rabbit secondary antibody were purchased from ProteinTech (Chicago, USA); the Alexa Fluor 555-conjugated goat anti-rabbit secondary antibody was purchased from Jackson ImmunoResearch (Pennsylvania, USA); 4′, 6-diamidino-2-phenylindole (DAPI), 5-diphenyltetrazolium bromide (MTT), and dimethyl sulfoxide (DMSO) were purchased from Sigma (Missouri, USA); TRIzol reagent used for total RNA extraction was purchased from Invitrogen (California, USA); 2× PCR SuperMix and SYBR Green Master Mix were purchased from Roche (Mannheim, Germany); keratinocyte serum-free medium (K-SFM) for the culture of human keratino-cytes was purchased from Gibco (California, USA); ELISA kits for the measurement of IL-1β and IL-18 secretion were purchased from R&D Systems (Minnesota, USA).

### 2.2 Cell Culture

As in our previous experiment [20, 22], the same method has been adopted this time, and an immortalized human oral mucosal epithelial cell line (Leuk-1) has been used in this study. This cell line was a generous gift from Professor Li Mao at the Department of Oncology and Diagnostic Sciences, University of Maryland Dental School, Baltimore, MD, USA. The cell line was expanded and passaged in K-SFM. The medium was supplemented with bovine pituitary extract (25 μg/mL), epidermal growth factor (0.2 ng/mL), and CaCl_2_ (0.4 mM). The passaged cells were cultured in 37℃ incubators in humidified air with 5% CO_2_. Cells were routinely grown to 70% confluence and trypsinized with 0.25% trypsin solution.

### 2.3 Organisms

Similar to our previous experiment [21, 23], *C. albicans* strain SC62342 was obtained from the Department of Mycology, Institute of Dermatology, Chinese Academy of Medical Sciences. The organisms were routinely propagated using yeast peptone dextrose agar (Difco Laboratories, Detroit, MI, USA) at 25℃.

### 2.4 Cigarette Smoke Extract (CSE) Preparation

The preparation of CSE was as previously described by our research group [7, 21, 24]. The Kentucky 3R4F research-reference filtered cigarettes used for CSE preparation were purchased from the Tobacco Research Institute, University of Kentucky (Lexington, KY, USA). The smoke from one 3R4F reference cigarette was bubbled with a peristaltic pump to 10 mL of keratinocyte serum-free medium at a speed of 50 mL/min. In order to remove bacteria and large particles, the solution was filtered through a 0.22-μm pore filter. The CSE solution, considered to be 100% CSE, was adjusted to a pH of 7.45 and used within 15 min after preparation.

### 2.5 MTT Assay

Cell viability was measured with a conventional MTT assay as previously described by our research group [23]. Leuk-1 cells were cultured in 96-well plates at a density of 1 × 10^5^ cells/mL, and were then co-cultured with CSE (0, 4%, 8%, and 16%) for different times (3, 5, and 7 days), and *C. albicans* with different proportions (Leuk-1 cells: *C. albicans* spores were 1:16, 1:4, 1:1, 4:1, and 16:1) for different times (0h, 3h, 6h, 9h, 12h, and 24h). Ten microlitres of MTT solution (5 mg/mL in phosphate-buffered saline) was added to each well. After incubation for 4 h, the supernatant was discarded, 150 microlitres of DMSO was added, and the plate was shaken to dissolve the formazan. The absorbance at a wavelength of 570 nm was measured by using a multiplate reader (Bio-Rad 680, Hercules, CA, USA).

### 2.6 Morphological Observation of *C. albicans*

On the basis of preliminary experiments [23], for this part, cells were divided into three groups: *C. albicans*, Leuk-1+ *C. albicans*, and Leuk-1+CSE+ *C. albicans*. Leuk-1 cells were cultured overnight in 96-well plates at a density of 2×10^5^ cells/mL. The following day, the medium was discarded, and the Leuk-1 cells were stimulated with 4% CSE or fresh medium (control) for 3 d. Next, *C. albicans* cells were counted in a haemo-cytometer and further adjusted to their final concentrations in complete K-SFM before addition to Leuk-1 cells. Leuk-1 cells were challenged with suspensions of *C. albicans* at a cell-to-*C. albicans* ratio of 4:1 based on MTT experiments. Before *C. albicans* challenge, one well of Leuk-1 cells was counted with a haemocytometer. Unchallenged Leuk-1 cells were used as the control group. *C. albicans* germ tube formation was assessed every 1 h, and *C. albicans* microcolony formation in each group was assessed after 24 h of co-culture.

### 2.7 Co-Culture of Leuk-1 and *C. albicans* Cells

To explore how cigarette smoke and *C. albicans* affect the oral mucosal defense, cells were divided into five groups: Leuk-1 cells; the Leuk-1+CSE group; the Leuk-1+ *C. albicans* group; the Leuk-1+CSE+*C. albicans* group, and the last group was the counting group. As mentioned above, Leuk-1 cells were cultured overnight in 6-well plates at a density of 1×10^5^ cells/mL. The rest of the steps were performed as described above. Samples of different types were collected according to the *C. albicans* culture time, and the details are shown below.

### 2.8 Immunofluorescence

As described in our previous experiment [7], Leuk-1 cells were grown on glass coverslips and stimulated with 4% CSE or fresh medium (control) for 3 d. Then, *C. albicans* cells were added and cultured for 4 hours. Next, the Leuk-1 cells were washed with PBS and fixed with 4% paraformaldehyde for 15 min at room temperature. After being washed in PBS, cells were permeabilized in 0.5% Triton X-100 in PBS, washed, and blocked with 5% BSA in PBS-0.1% Tween 20 for 1 h at 37℃. Next, the cells were incubated with primary antibodies overnight at 4℃. Primary antibodies against the following proteins or organisms were used: NLRP3 (1:200), caspase-1 (1:200), IL-1β (1:25), IL-18 (1:100), and *C. albicans* (1:100). The next day, coverslips were washed with PBS, and were then incubated with FITC-conjugated goat anti-rabbit, Cy3-conjugated goat anti-mouse or Alexa Fluor 555-(red)-conjugated goat anti-rabbit secondary antibodies for 1 h at room temperature. To stain nuclei, DAPI was added for 5 min, and the slides were examined under a confocal laser scanning microscope (FluoView FV10i, Olympus, USA).

### 2.9 Quantitative Real-time PCR (Q-PCR)

Leuk-1 cells were stimulated with 4% CSE or fresh medium (control) for 3 d, and *C. albicans* cells were then added and cultured for 12 h. RNA was isolated from all samples by using TRIzol reagent, and then cDNA was produced from total RNA by reverse-transcription reaction. Q-PCR analyses were performed using SYBR Green Q-PCR kit (Roche, Mannheim, Germany) in a 7300 Real-Time PCR System (Applied Biosystems, Waltham, MA, USA). The primer pairs targeting the mRNAs were as follows: NLRP3 forward, CTA GCC ACG CTA ATG ATC GAC TT, and reverse, CAG TAA ACC CAT CCA CTC CTC TTC; caspase-1 forward, AGG GAC GCT GGG ACT CTC, and reverse, AAG CTT GAC ATT CCC TTC TGA G; IL-1β forward, ATG CAC CTG TAC GAT CAC TGA, and reverse, ACA AAG GAC ATG GAG AAC ACC; and IL-18 forward, CAG ACC TTC CAG ATC GCT TC, and reverse, CCC CCA ATT CAT CCT CTT TT.

### 2.10 Enzyme-linked Immunosorbent Assay (ELISA)

To detect the levels of IL-1β and IL-18 released by Leuk-1 cells, we collected and quantified the cell culture supernatants with enzyme-linked immunosor-bent assay kits according to the manufacturer’s instructions. The absorbance at 450 nm was read using a spectrophotometric plate reader.

### 2.11 Statistical Analysis

Two-way analysis of variance (ANOVA) or least significant difference (LSD) test were used to compare all individual groups. The data are presented as mean ± SEM. The analysis was performed using GraphPad Prism (version 5.0). All statistical analyses were 2-sided, and differences for which *P* <0.05 were considered statistically significant.

## 3 RESULTS

### 3.1 Effects of CSE and *C. albicans* on Leuk-1 Cells' Viability

To detect the effect of CSE and *C. albicans* on the viability of Leuk-1 cells in coculture at different concentrations and different times, an MTT assay was performed. As shown in Fig. (**2A**). the result found that the cell viability of Leuk-1 cells treated with 4% CSE and 8% CSE showed no significant change (*P*>0.05). However, when it reached 16% CSE, the cell growth was significantly inhibited (*P*<0.05). As shown in Fig. (**2B**). at the cell-to-*C. albicans* ratios of 16:1 and 4:1, cocultivation with *C. albicans* for 24 h remarkably reduced the cell viability of Leuk-1 cells. At the ratio of 1:1, the cell viability was clearly decreased when cocultivated with *C. albicans* for 12 and 24 h. Then, statistically notable alterations of cell viability were observed at the ratio of 1:4 or 1:16 for 3, 6, 9, 12, and 24 h.

### 3.2 CSE Eliminated the Suppressive Effects of Leuk-1 Cells on *C. albicans* Formation

**Fig. (3):**
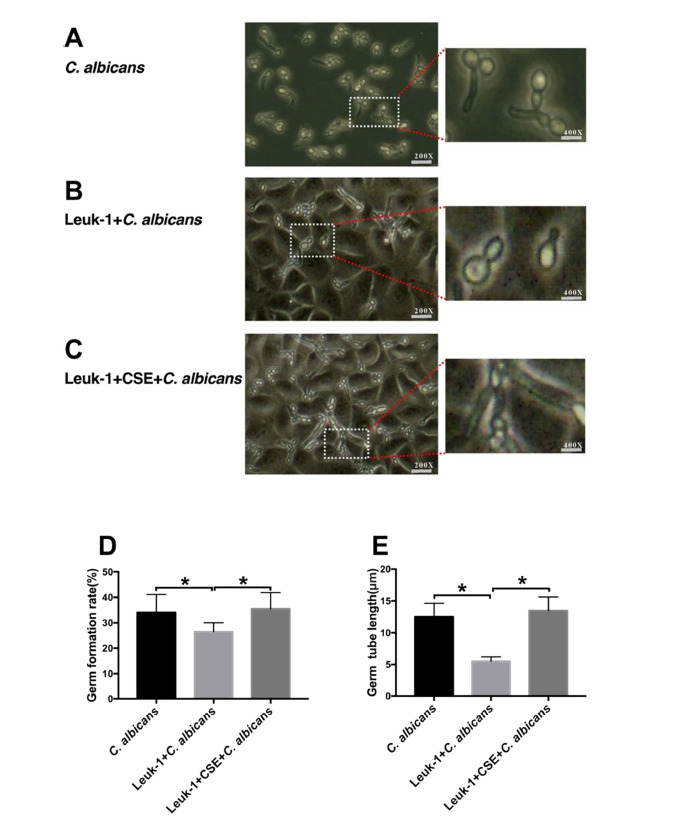
The alterations in *C. albicans* germ tube formation after co-culture with Leuk-1 cells and treatment with CSE. Microscopic observation of germ tubes at 200× magnification after cocultivation of *C. albicans* cells with Leuk-1 for 2 h: (**A**) *C. albicans,* (**B**) Leuk-1+ *C. albicans,* (**C**) Leuk-1+CSE+*C. albicans.* (**D**) The germ tube formation rate in the three groups. (**E**) The germ tube lengths in the three groups. The results are from three independent experiments (*P < 0.05. Insets: 400× original magnification).

**Fig. (4):**
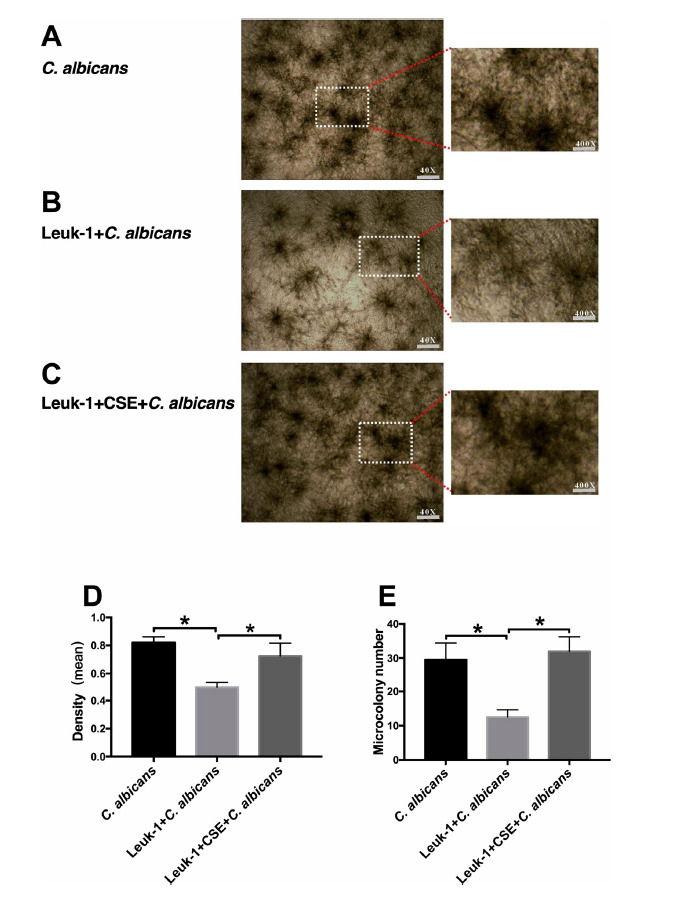
The alterations in *C. albicans* microcolony formation after co-culture with Leuk-1 cells and treatment with CSE. Microscopic observation of microcolony formation after cocultivation of *C. albicans* and Leuk-1 cells for 24 h at 40× magnification: (**A**) *C. albicans,* (**B**) Leuk-1+ *C. albicans,* (**C**) Leuk-1+CSE+ *C. albicans.* (**D**) The optical density values of microcolony images in the three groups. (**E**) The numbers of microcolonies in the three groups. The results are from three independent experiments (*P < 0.05. Insets: 400× original magnification).

As shown in (Fig. **3A**-**E**). after *C. albicans* cells were added for 2 h, a comparison of the germ tube formation rate in each group at 200× magnification revealed that the germ tube formation rate in the Leuk-1+CSE+*C. albicans* group was higher than that in the Leuk-1+ *C. albicans* group (*P* <0.05) and the germ tube formation rate in the *C. albicans* group was higher than that in the Leuk-1+ *C. albicans* group (*P* <0.05). The same trend applied to the germ tube lengths in the three groups. As shown in (Fig. **4A**-**E**). after *C. albicans* cells were added for 24 h, a comparison of the optical density values and images showing the colony numbers in the three groups at 40× magnification indicated that the colony density in the Leuk-1+CSE+ *C. albicans* group was higher than that in the Leuk-1+ *C. albicans* group (*P* <0.05) and the colony density in the *C. albicans* group was higher than that in the Leuk-1+ *C. albicans* group (*P* <0.05). The same trend applied to the numbers of microcolonies in the three groups. These results seem to indicate that Leuk-1 cells could inhibit the growth of *C. albicans*; however, CSE promoted the proliferation of *C. albicans* cells by reducing the organism’s resistance to *C. albicans* infection.

### 3.3 Suppressive Effects of CSE and Activating Effects of *C. albicans* on the NLRP3 Inflammasome and its Downstream Effectors in Leuk-1 Cells

To explore the mechanism of CSE or *C. albicans* exposure on oral epithelial cells, we tested the secretion of inflammasome–dependent cytokines; both the mRNA and protein levels of NLRP3 inflammasome pathway were found to be significantly reduced *in vitro* in the presence of CSE. As shown in Fig. (**5A**-**5D**). according to Q-PCR analysis, compared to those in the control group, the expression levels of NLRP3, caspase-1, IL-1β, and IL-18 were decreased in the CSE treatment group (*P* <0.05) and increased in the *C. albicans* group (*P* <0.05). Inclusion of CSE to the *C. albicans*-containing medium significantly reduced the levels of NLRP3, caspase-1, IL-1β, and IL-18, compared to those in the *C. albicans* group (*P* <0.05). A similar tendency was observed in the cell culture supernatant Fig. (**5E**-**5F**). the cell density was not significantly different between the groups, and the release of IL-1β and IL-18 was markedly repressed by the addition of CSE. To achieve more robust evidence, we conducted a series of immunofluorescence studies Fig. (**6A** and **B**). and consistent with Q-PCR and ELISA analysis, we also noted that the elevated expression levels of NLRP3, caspase-1, IL-1β, and IL-18 in *C. albicans* group were markedly reduced by CSE and *C. albicans* infection. These data demonstrate that CSE reduces inflammation by repressing *C. albicans*-mediated activation of the NLRP3 inflammasome.

## 4 DISCUSSION

To date, it is clear that several risk factors are involved in the development of oral diseases, and the most common and established of these risk factors is smoking. Furthermore, *C. albicans* has been implicated in the pathogenesis of oral premalignant lesions. As a polymorphic fungus, the ability of *C. albicans* to undergo morphological transformation from yeast to germ tube to hyphae is considered its central virulence attribute [25]. At distinct stages of *C. albicans* infection to the epithelium, both of the two most important morphologies, the germ tube and hyphal forms, are required for virulence [26]. The development of these forms is also one of the indicators used to evaluate the effect of drugs or other factors on fungal growth [27, 28]. In our morphological experiment, Leuk-1 cells inhibited *C. albicans* germ tube and microcolony formation in comparison to that in the control group, and the addition of CSE treatment promoted the growth of *C. albicans*. These results indicate that smoking may weaken the defense ability of oral epithelial cells and promote the growth of *C. albicans,* similar to the effects observed in our previous animal studies [21]. Subsequently, we further explored the underlying mechanisms. A previous study indicated that cigarette smoke can cause functional and structural alterations in oral epithelial cells, contributing to the decrease in antifungal activity and facilitating the proliferation of *C. albicans* [29]. The results of another study supported the hypothesis that cigarette smoke may have an indirect effect on *C. albicans* by enhancing glycosylation reactions in the mucosal epithelium [30].

As a common PRR in oral epithelial cells, the NLRP3 inflammasome plays an essential role in innate immunity. Our results show that the NLRP3 signal pathway that protects the oral mucosa from *C. albicans* infection is compromised by cigarette smoke, which is consistent with the findings of our previous animal study [21]. Emerging evidence indicates that the NLRP3 inflammasome plays an indispensable role in the defense response to *Candida* infection. Studies have shown that NLRP3-deficient mice infected with *C. albicans* display lower serum IL-1β levels, reduced survival, higher fungal burdens, and higher clinical severity scores than wild-type mice [31, 32]. Our results indicate that *C. albicans* activates the NLRP3 signal pathway. However, the expression of key molecules in the pathway was suppressed after treatment with CSE, indicating that cigarette smoke can inhibit the innate immune defense of oral epithelial cells. Similar to our study, other studies have shown that cigarette smoke represses central components of the innate immune response [33]. Based on this observation, a recent investigation confirmed that CSE decreases the protein abundance of NLRP3 and secretion of IL-1β and IL-18 through increased ubiquitin-mediated proteasomal processing in monocytes and macrophages [34]. Another experiment showed that cigarette smoke inhibits the NLRP3 inflammasome and leads to caspase-1 activation *via* the TLR4-TRIF-caspase-8 axis in human macrophages [34]. Our findings add to a growing body of literature indicating that cigarette smoke impairs the immune surveillance of oral epithelial cells. Our results suggested that in oral epithelial cells, NLRP3 inflammasome might be one of the target pathways through which CSE attenuates innate immunity and leads to oral disorders, including oral leukoplakia.

Our study has some limitations that need to be improved in future experiments, for example, whether other forms of tobacco (such as chewing tobacco) might impact the oral mucosa in a similar way, or how to combine closely our CSE *in vitro* experiments with clinical smokers' oral mucosa.

In summary, the present study suggested that cigarette smoke suppresses the innate immune response of oral epithelial cells and the defense response against *C. albicans via* the NLRP3 signal pathway. Cigarette smoke exposure might synergize with *C. albicans* infection in the progression of oral disorders. The knowledge and understanding of the effects of cigarette smoke and *C. albicans* on the innate immune response of oral epithelial cells offer novel insight into potential therapeutic strategies for oral disorders. Further investigations of the exact mechanism and functional verification are needed to clarify the synergistic effect of smoking and *Candida* and the exact role of the NLRP3 inflammasome in the pathogenesis of oral disorders.

## CONCLUSION

Our study suggested that CSE weakened the immune defense ability of Leuk-1 cells and promoted the formation of germ tube and colony, which accelerated the invasion of *candida albicans* on Leuk-1 cells. Further, NLRP3 inflammasome may be one of the ways in which CSE regulates innate immunity of oral mucosa in oral mucosal epithelial cells mechanistically.

## Figures and Tables

**Fig. (2):**
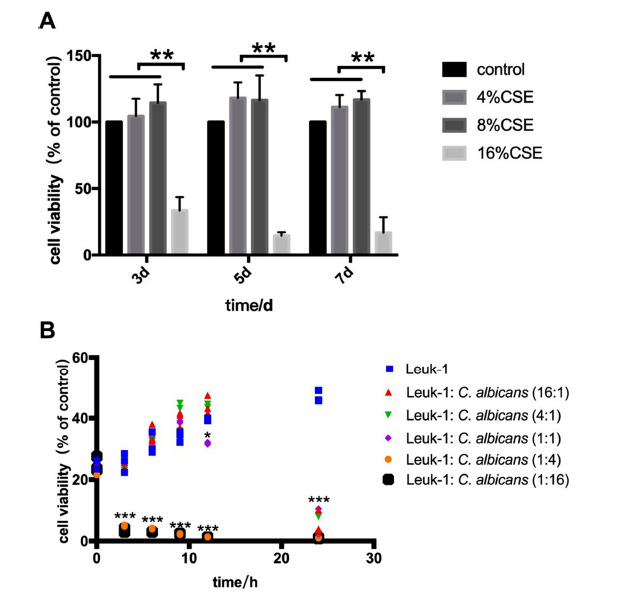
Effects of CSE **(A)** and *C. albicans* **(B)** infection on the cell viability of Leuk-1 cells according to MTT. (**A**) Leuk-1 cells were treated with different concentrations (4%, 8%, and 16%) of CSE for 3, 5, and 7 d. The cell viability of Leuk-1 cells treated with 4% CSE and 8% CSE for 3, 5, and 7 d showed no significant change (P>0.05). Then, the cell growth was significantly inhibited with 16% CSE (*P*<0.05). Statistical significance: **P < 0.01 *vs*. cells without CSE coculture at the same time point. (**B**) Leuk-1 cells were treated with different ratios of *C. albicans* for 3, 6, 9, 12, and 24 h. At the cell-to-*C. albicans* ratios of 16:1 and 4:1, cocultivation with *C. albicans* for 24 h remarkably reduced the cell viability of Leuk-1 cells. When it reached 1:1, the cell viability was decreased clearly for 12 and 24 h. Then, statistically notable alterations of cell viability were observed at the ratio of 1:4 or 1:16 for 3, 6, 9, 12, and 24 h. The results are from three independent experiments. Statistical significance: *P < 0.05, ***P < 0.001 *vs*. cells without *C. albicans* coculture at the same time point.

**Fig. (5):**
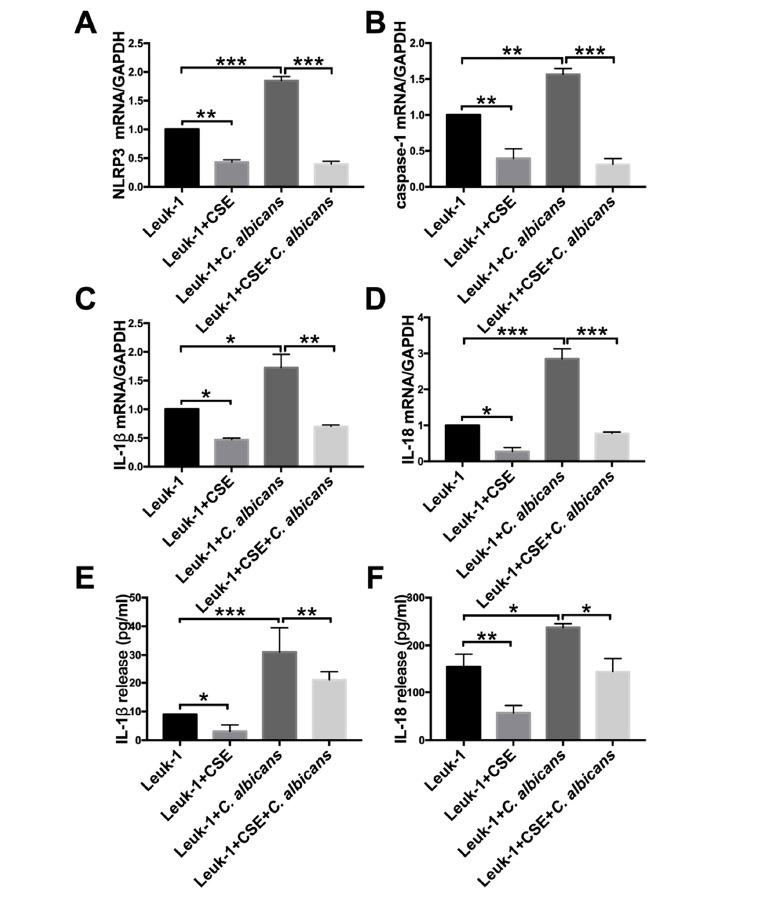
Q-PCR (**A-D**) and ELISA (**E-F**) assay of NLRP3 inflammasome and its downstream effectors after CSE treatment and *C. albicans* challenge. A significant decrease was observed in the expression of NLRP3, caspase-1, IL-1β, and IL-18 in the CSE*-*infected group. In contrast, the *C. albicans-*infected group showed an increase in expression relative to the control group. Reduced levels of the NLRP3 inflammasome components were observed in the coculture group compared to that of the *C. albicans* treatment alone. The results are from three independent experiments (*P < 0.05, **P < 0.01, ***P < 0.001).

**Fig. (6):**
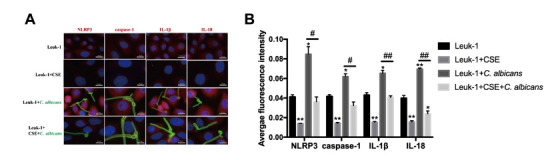
Confocal microscopy analysis of NLRP3 inflammasome components in response to treatment with CSE and *C. albicans*. (**A**) Merged images were obtained and analysed using an Olympus confocal microscope. Fluorescence images were acquired in the green channel at 555 nm with excitation at 495 nm or in the red channel at 590 nm with excitation at 560 nm. Blue fluorescence indicates nuclear staining (magnification: 400× for staining, scale bar: 50 μm). (**B**) Average fluorescence intensity values for the staining images in panel A. We noted that the elevated expression levels of NLRP3, caspase-1, IL-1β, and IL-18 in the *C. albicans* group were markedly reduced by CSE and *C. albicans* infection. The results are from three independent experiments (*P < 0.05 compared to the control group; **P < 0.01 compared to the control group; ***P < 0.001 compared to the control group; #*P* < 0.05 compared to the *C. albicans* group).

## Data Availability

All the data and supportive information are provided within the article.

## LIST OF ABBREVIATIONS

ANOVA: Two-way Analysis of Variance
CSE: Cigarette Smoke Extract
DAMPs: Danger-Associated Molecular Patterns
LSD: Least Significant Difference
PAMPs: Pathogen-Associate Molecular Patterns
PRRs: Pattern Recognition Receptors
